# Environmentally Friendly Approach for Nd_2_Fe_14_B Magnetic Phase Extraction by Selective Chemical Leaching: A Proof-of-Concept Study

**DOI:** 10.3390/ma16145181

**Published:** 2023-07-23

**Authors:** Sina Khoshsima, Janja Vidmar, Zoran Samardžija, Tomaž Tomše, Monika Kušter, Amit Mishra, Sašo Šturm, Kristina Žužek

**Affiliations:** 1 Department for Nanostructured Materials, Jožef Stefan Institute, Jamova 39, SI-1000 Ljubljana, Slovenia; zoran.samardzija@ijs.si (Z.S.); tomaz.tomse@ijs.si (T.T.); monika.kuster@ijs.si (M.K.); amit.mishra@ijs.si (A.M.); saso.sturm@ijs.si (S.Š.); tina.zuzek@ijs.si (K.Ž.); 2 Department of Environmental Sciences, Jožef Stefan Institute, Jamova 39, SI-1000 Ljubljana, Slovenia; janja.vidmar@ijs.si; 3 Jožef Stefan International Postgraduate School, Jamova 39, SI-1000 Ljubljana, Slovenia

**Keywords:** rare earths, neodymium magnets, magnetic properties, leaching, organic acid, environmentally friendly

## Abstract

The green transition initiative has exposed the importance of effective recycling of Nd-Fe-B magnets for achieving sustainability and foreign independence. In this study, we considered strip-cast, hydrogenated, jet-milled Nd-Fe-B powder as a case study to explore the potential for selective chemical leaching of the Nd-rich phase, aiming to extract the Nd_2_Fe_14_B matrix phase. Diluted citric and nitric acids at concentrations of 0.01, 0.1, and 1 M were considered potential leaching mediums, and the leaching time was 15 min. Microstructural investigation, magnetic characterization, and elemental compositional analysis were performed to investigate leaching efficiency and selectivity. Based on SEM analysis, Nd/Fe ratio monitoring via ICP-MS, and the high moment/mass value at 160 emu/g for the sample leached with 1 M citric acid, 1 M citric acid proved highly selective toward the Nd-rich phase. Exposure to nitric acid resulted in a structurally damaged Nd_2_Fe_14_B matrix phase and severely diminished moment/mass value at 96.2 emu/g, thus making the nitric acid unsuitable for selective leaching. The presence of hydrogen introduced into the material via the hydrogen decrepitation process did not notably influence the leaching dynamics. The proposed leaching process based on mild organic acids is environmentally friendly and can be scaled up and adopted for reprocessing industrial scrap or end-of-life Nd-Fe-B magnets to obtain single-phase Nd-Fe-B powders that can be used for novel magnet-making.

## 1. Introduction

Due to their distinctive physical and chemical properties, rare earth elements (REE) are facing a rise in demand in a variety of applications such as permanent magnets, catalysts, NiMH batteries, electric (EV) and hybrid electric vehicles (HEV), and generators used in wind turbines [1]. The REE-containing products in the electric mobility or green energy production industry are neodymium-iron-boron or samarium-cobalt permanent magnets (Nd-Fe-B or Sm-Co, respectively). Nd-Fe-B magnets are especially desirable and essential for the EV and HEV industry due to their outstanding energy product ((BH)_max_), with a theoretical maximum of 512 kJ/m^3^. Consisting of approximately 31 wt.% REEs (mostly Nd), 66 wt.% Fe, and around 1 wt.% B, Nd-Fe-B production scrap and end-of-life (EoL) magnets are considered a legitimate secondary source for REE recovery and recycling [2]. There have been efforts to process and re-evaluate EoL NdFeB magnets [3]. The conventional industrial method for recycling REEs from Nd-Fe-B magnets in their elemental form is hydrometallurgy due to its high percentage of recovery and selectivity. Hydrometallurgical leaching of REEs has some advantages compared to pyrometallurgical methods, such as the low energy consumption that arises from its lower operation temperature range (e.g., 27–110 °C) [4,5,6]. Pyrometallurgical methods are known to have a larger environmental footprint in harmful gas emissions. Although quantitative results are difficult to obtain, a study reports detection of CO, CO_2_, and HF as the gas by-products of the incineration step of the pyrolysis process, which are extremely harmful for the environment and life (e.g., HF and CO) or are the major cause of global warming (e.g., CO_2_) [7]. Hydrometallurgical processes are also more flexible in terms of adaptation of leaching parameters (e.g., change of the leaching reagent, time of leaching, and concentration of the acidic medium used for leaching), which facilitates possible changes in the process as technology evolves [8]. One study reported >95% recovery of REEs via the formation of a sulfate mixture using NdFeB powder and concentrated H_2_SO_4_ (12–16 mol/L acid concentrations and leaching times of 15 min–24 h) and post-processing [9]. Another group investigated different acid concentrations and leaching temperature effects on the most optimal recovery of REEs using H_2_SO_4_ [10]. The optimal conditions were found to be 70 °C and 3 mol/L H_2_SO_4_ with a leaching time of 4 h [10]. The effect of different leaching agents was studied by Lee et al., using HCl, H_2_SO_4_, HNO_3,_ and NaOH as leaching agents, revealing that HCl and H_2_SO_4_ are the best choices for leaching agents [11]. However, these methods are still based on using a significant quantity of strong and concentrated mineral acids, leading to adverse environmental circumstances. These include the release of toxic gasses and acidification of soil during the neutralization of the waste, which requires additional purification steps to de-acidify the soil to reduce environmental damage [12,13,14].

Nd-Fe-B sintered magnets are multiphase materials. As shown by the phase diagram of the Nd-Fe-B ternary system [15], magnets consist primarily of the hard-magnetic Nd_2_Fe_14_B phase (85–87 volume %) and secondary Nd phase (10–12 volume %) [16]. However, as a result of minor alloying additions, the material’s phase composition and chemistry of the phases are more intricate [17]. For easier interpretation of the results presented in this study and following the often-used terminology when describing the microstructure of Nd-Fe-B magnets, the terms “Nd_2_Fe_14_B matrix” and “Nd-rich phase” are used throughout this paper. The Nd-rich phase is required for liquid-phase sintering and is a prerequisite for grain-boundary structure refinement and high-temperature magnetic performance. When compromised by external stimuli such as air or water, magnetic properties irreversibly deteriorate, and such a magnet is considered an EoL magnet.

On the other hand, the Nd_2_Fe_14_B grains are generally intact at this point and can be reused as primary building blocks for new sintered magnets. Building on this idea, our group introduced a procedure where instead of leaching all the chemical elements out of the scrap/EoL magnets, a selective electrochemical leaching method was devised. It was shown that the Nd-rich phase could not be leached out from the complex Nd-Fe-B microstructure. The Nd-rich phase is etched away preferentially because of the more negative electrochemical potential [18,19,20], resulting in the separation and exertion of the Nd_2_Fe_14_B matrix phase grains [18]. However, achieving sustainability and total industrial-level yield out of this method has proven difficult for the moment and requires more technical inspection and optimization, given the meager experimental yields. 

Here, an alternative selective leaching approach based on acid leaching is introduced. Considering the work of Gergoric et al., who showed that the kinetics of fully dissolving EoL Nd-Fe-B magnets in citric acid is much slower than mineral acids [21], the present study aimed to selectively leach away the Nd–rich phase using this mild acid without compromising the structure and magnetic properties of the Nd_2_Fe_14_B matrix phase. A jet-milled microcrystalline Nd-Fe-B powder, produced from strip-cast and hydrogen-decrepitated alloy, the feedstock for sintered Nd-Fe-B magnets, was used as a case study instead of EoL magnets to achieve higher yields. The influence of the acid type (citric or nitric) and acid concentration on the leached powders’ microstructure, magnetic properties, and elemental composition was investigated. As the hydrogen processing of magnetic scrap (HPMS) to obtain a friable coarse powder for further use is one of the crucial technologies in the ‘’green’’ recycling of EoL magnets [22], the effect of hydrogen present in the material’s structure on the leaching efficiency was also studied. Results of the investigation confirmed that the diluted citric acid selectively leaches away the Nd-rich phase, and the impact of the hydrogen on the leaching efficiency was minor. 

## 2. Materials and Methods

### 2.1. Material Characterization

The Nd-Fe-B-type strip-cast alloy with a nominal composition of Nd_30.1_Pr_0.6_Dy_1_Fe_63.85_Co_3_Ga_0.22_Cu_0.15_Al_0.15_B_0.93_ (wt.%) was hydrogen-decrepitated (HD) and jet-milled (JM) at Magneti Ljubljana d.d. 

To investigate the effect of hydrogen on leaching efficiency, a portion of the jet-milled powder was thermally degassed at 600 °C for 20 min under vacuum. The evolution of pressure with temperature was investigated by heating the powder to 1000 °C with a 2 °C/min heating rate in a tube furnace (Nabertherm Gmbh R50/250/13, Lilenthal, Germany) connected to a vacuum pump (Pfeiffer vacuum Gmbh, Aßlar, Germany). The two powders are hereafter referred to as jet-milled not degassed (JM-NDG) and jet-milled degassed (JM-DG). The optimal conditions for degassing the JM-NDG powder were investigated under an initial vacuum of 1 × 10^−5^ mbar. Figure S1 (Supplementary Information) displays the pressure evolution with temperatures up to 1000 °C. Two distinctive peaks are observed that indicate a two-step desorption of hydrogen. According to the literature, the first peak centered at ≈150 °C is associated with hydrogen desorption from the matrix phase (Nd_2_Fe_14_B) and partial desorption from the secondary phase (Nd-rich phase). In comparison, the second peak at ≈575 °C signifies complete desorption from the secondary phase [23]. Based on this result, the JM-NDG powder was degassed at 600 °C to obtain a hydrogen-free powder (JM-DG) for further processing.

The microstructure of the samples before and after leaching was investigated using a Thermo Fisher QUANTA 650 scanning electron microscope operating under high vacuum equipped with Energy Dispersive X-ray Analysis (EDS) Oxford Instruments Aztec with Ultim Max 40 mm^2^ SDD sensor. To do so, the samples were embedded in resin, and the surface of the prepared resin mold was polished with emery paper with grades of 500, 900, 1200, and 2000 in a sequence and finally polished with diamond paste for a smooth finish and then cleaned with ethanol. The untreated (i.e., not leached with acid) JM-NDG and JM-DG powders were analyzed via Empyrean multipurpose diffractometer within the 10–90° 2theta (2θ) range with a step size of 0.01/s to monitor the presence of different phases (e.g., hydrides, oxides, etc.) within the microstructure. Given the reactive nature of the samples, the measurements were carried out using a PANalytical PEEK dome and prepared inside a glovebox. 

### 2.2. Leaching Procedure

The citric acid (Sigma-Aldrich, Saint Louis, MO, USA, ≥99.5%) and nitric acid 67–69% (Carlo Erba Reagents, Cornaredo, Italy) were used as leaching agents. The leaching solutions were prepared by diluting the acids with Milli-Q water (18.2 MΩ cm) obtained from the Direct-Q 5 Ultrapure aqueous system (Merck Millipore, Milford, MA, USA) to concentrations of 0.01, 0.1, and 1 mol/L. The S:L ratio was kept constant at 1:15 (1 g of a powder to 15 mL of diluted acid) for 15 min. All experiments on JM-NDG and JM-DG were performed at room temperature in beakers without any additional agitation force (e.g., stirring, vibration, etc.) and in triplicate for statistical reliability. The leached powders were washed with deionized water and ethanol and filtered and dried under a vacuum. 

### 2.3. Magnetic Measurements

The hysteresis loops of all powder samples before and after leaching were measured, and the maximum measured magnetic moment per unit mass value (emu/g) was accepted as the determining magnetic property. The characterization was carried out using a Lakeshore 8600 series vibrating sample magnetometer (VSM) by fixating samples in specialized holders and under a field of 2 Tesla (T). 

### 2.4. Determination of the Chemical Composition of Powder Samples via ICP-MS

#### Sample Preparation

Approximately 0.1 g of JM-NDG and JM-DG powders before and after leaching with 1 M citric and nitric acids were weighed into a polypropylene tube, and 8 mL of 1 M nitric acid, prepared from 67–69% HNO_3_ (Carlo Erba Reagents, Italy), and 2 mL of 1 M hydrochloric acid, prepared from 30% HCl (Merck KGaA, Darmstadt, Germany), were added. After closing the tube, the sample was shaken. Then, 2 mL of concentrated nitric acid (15.2 M HNO_3_) and 2 mL of concentrated hydrochloric acid (11.4 M HCl) were added, and the sample was left in the closed tube overnight at room temperature. The next day, the resulting clear yellow solution was diluted with 1% nitric acid prior to ICP-MS analysis. Each sample was digested in duplicate. A blank sample was prepared by adding 0.1 g of MilliQ water instead of a powder sample and subjected to the same digestion procedure as described above. Blank values were subtracted from the values of the samples.

### 2.5. ICP-MS Analysis

Elemental compositions were determined via inductively coupled plasma mass spectrometry (ICP-MS). An Agilent 7700x Series ICP-MS instrument (Agilent Technologies, Tokyo, Japan) was equipped with an autosampler (ASX-500, Agilent Technologies), a Teflon Mira Mist nebulizer, and a double-spray, Scott-type spray chamber made of quartz. The ICP-MS instrumental parameters summarized in Table S1 (Supplementary Information) were optimized for the best sensitivity and robustness. Quantification was performed based on the external calibration by measuring standards containing elements of interest in the concentration range of 0.1–1000 µg L^−1^ with online internal standardization (25 µg L^−1^ solution of Rh and Ir). Calibration standard solutions were prepared in 1 % nitric acid from stock solutions of ICP multi-element standard solution IV (1000 µg mL^−1^ Al, B, Fe, Co, Cu, Ga in 6.5% HNO_3_, obtained from Merck) and rare earth element mix for ICP (50 µg mL^−1^ of Pr, Nd and Dy in 2 % HNO_3_, obtained from Fluka Analytical, Sigma-Aldrich, St. Louis, MO, USA).

## 3. Results and Discussion

### 3.1. X-ray Diffraction Analysis of Non-Leached Nd-Fe-B Powders

**Figure 1:**
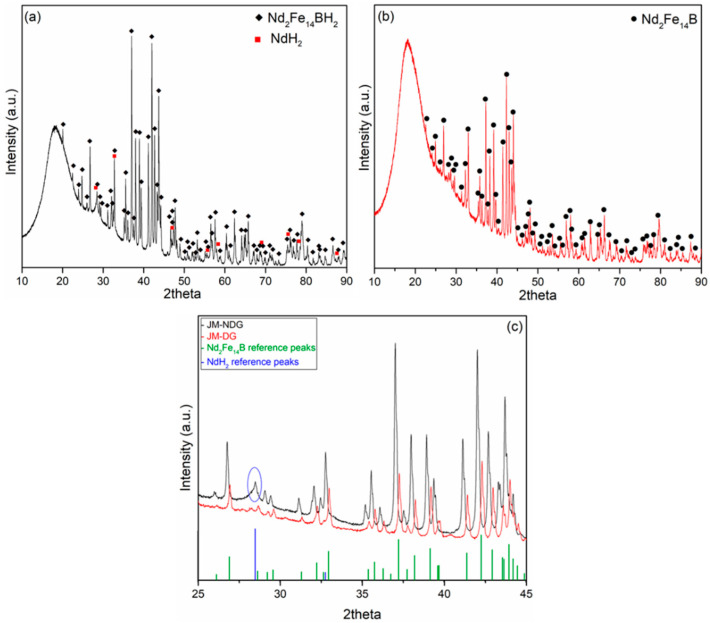
XRD pattern of the (**a**) jet-milled not degassed (JM-NDG), (**b**) jet-milled and degassed (JM-DG) Nd-Fe-B powder, (**c**) focused area comparison (25 ≤ 2θ ≤ 45) of the two patterns against each other and the reference peaks of the Nd_2_Fe_14_B (ICDD Card No: 00-039-0473, Tetragonal) and NdH_2_ (ICDD Card No: 96-153-8931, Cubic).

The XRD patterns of the JM-NDG and JM-DG powders and their respective comparison against their corresponding reference peaks obtained from the International Crystallographic Diffraction Database (ICDD) are presented in Figure 1a–c. The bump observed in the 2theta range between 12 and 25 originates from the dome of the sample holder that was used to ensure a protective environment for the oxygen-sensitive powders. The pattern of the JM-NDG powder matches the Nd_2_Fe_14_BH_2_ (ICDD Card No: 04-012-8942, Tetragonal) and the NdH_2_ (ICDD Card No: 96-153-8931, Cubic) phases (Figure 1a). The pattern of the JM-DG powder matches the Nd_2_Fe_14_B phase (ICDD Card No: 00-039-0473, Tetragonal) (Figure 1b). Other than NdH_2_, no other secondary phases, e.g., Nd_2_O_3_, otherwise often found in the microstructure of Nd-Fe-B magnets, were detected via this analysis. Such minor phases are most likely below the detection limit of XRD (>2 volume %) [24]. Figure 1c exhibits a focused area comparison (25 ≤ 2θ ≤ 45) of the two patterns against each other and the reference peaks of the Nd_2_Fe_14_B and NdH_2_. The peaks corresponding to the Nd_2_Fe_14_BH_2_ phase (powder JM-NDG) shifted to lower angles compared to the Nd_2_Fe_14_B phase (powder JM-DG), signifying cell expansion due to interstitial hydrogen. Therefore, the XRD analysis confirms that the hydrogen was desorbed from the JM-NDG powder upon the thermal treatment. The exact hydrogen stoichiometry of phases formed during the hydrogen decrepitation process might differ from the stoichiometry specified in the PDF cards and cannot be deduced from this analysis [25]. The presence of a hydrogen-containing Nd-rich phase in the structure of the JM-NDG sample is evident from the broad peak positioned at 2θ ~ 28 encircled in blue. 

### 3.2. Microstructure Analysis of Non-Leached and Leached Nd-Fe-B Powders

**Figure 2:**
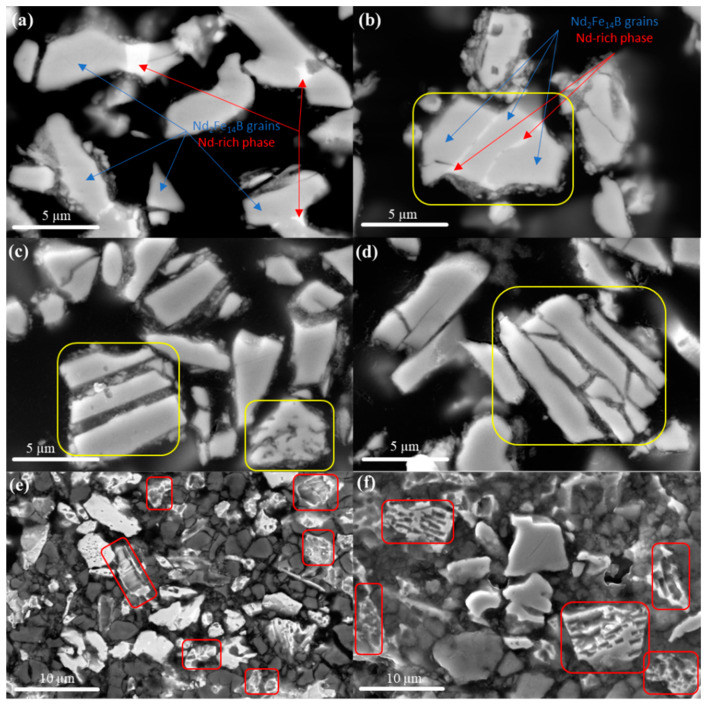
SEM images of the: (**a**) JM-NDG powder before acid treatment, (**b**) JM-DG powder before acid treatment, (**c**) JM-NDG after leaching with 1 M citric acid, (**d**) JM-DG after leaching with 1 M citric acid, (**e**) JM-NDG after leaching with 1 M nitric acid, (**f**) JM-DG after leaching with 1 M nitric acid.

Backscattered-electron (BSE) mode SEM images of the untreated powders (JM-NDG and JM-DG) and samples treated with citric and nitric acid are presented in Figure 2a–f. The dark contrast corresponds to the resin used to embed the powder particles for microstructural investigation. It is noted that the morphology of the JM-NDG and JM-DG powder particles (Figure 2a,b, respectively) is irregular, with an average particle size ranging between 4 and 7 µm. Note that this value is underestimated in the cross-sectional view and that the actual particle size is larger. Respective phases are observed through compositional contrast. Particles/grains display gray (indicated by blue arrows) or bright (indicated by red arrows) contrast. In Figure 2b, a polycrystalline particle consisting of grains with gray contrast, separated by a bright phase, is marked with a yellow rectangle. Such a phase distribution is typical of a strip-cast Nd-Fe-B alloy [26]. The EDS point analysis of the phases depicted by red and blue arrows revealed that the gray matrix phase is composed of Fe 84.3 at. % and Nd 12.5 at. %, corresponding to the 2–14 stoichiometry, thus confirming that it is the Nd_2_Fe_14_B phase. Boron, being a light element, was not identified.

In comparison, the bright phase consists of Nd (~30.7 at. %), Fe (~60.1 at. %), Co (~8.2 at. %), and Cu (~1.0 at. %), identifying it as an Nd-rich phase. However, considering the limitations of the EDS analysis method, which are due to the limited spatial resolution of the X-ray-generated interaction volume (about 1 µm^3^), determining the exact composition of the bright phases proves difficult. The microstructures of the JM-NDG and JM-DG powders after leaching with 1 M citric acid are shown in Figure 2c,d, respectively. Compared to the particle shown in Figure 2b, the strip-like Nd-rich phase is absent in the leached powder particles (marked with yellow rectangles in Figure 2c,d). Demonstrably, 1 M citric acid selectively leached away the Nd-rich phase. These findings are further supported by the magnetic characterization and ICP analysis results, as discussed later in the text. The effect on the microstructure of the JM-NDG and JM-DG powders of leaching with 1 M nitric acid is depicted in Figure 2e,f, respectively. In the sections highlighted by red rectangles, it can be observed that the 1 M nitric acid attacked not only the Nd-rich phase but also the Nd_2_Fe_14_B phase and was therefore, not selective enough to extract the Nd_2_Fe_14_B grains from the Nd-Fe-B powders intact. In short, SEM investigation revealed that the nature of the acid plays a significant role in the selectivity and efficiency of leaching. On the other hand, a comparison of Figure 2c,e (leached JM-NDG) with Figure 2d,f (leached JM-DG) reveals no apparent influence of hydrogen on leaching efficiency, as the microstructures of the samples leached under similar conditions are similar.

### 3.3. Effect of Leaching on the Magnetic Properties of Nd-Fe-B Powders

**Figure 3:**
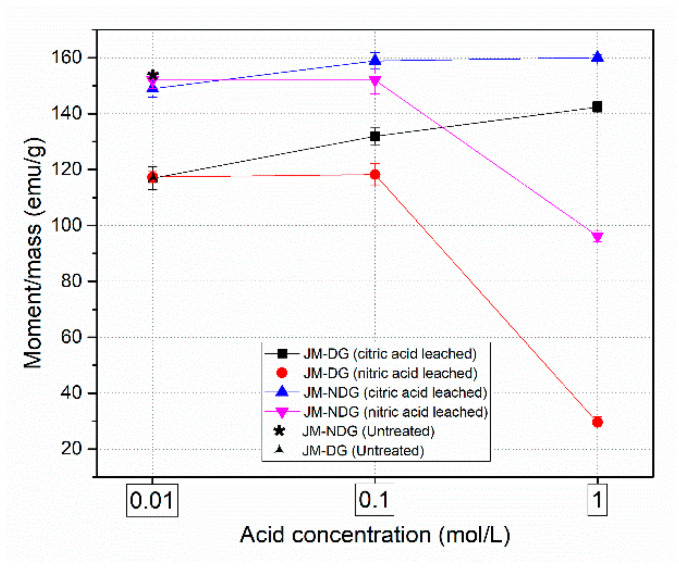
Effect of leaching with citric and nitric acids on the room-temperature magnetic properties of the JM-NDG and JM-DG powders.

The effect of leaching with nitric and citric acids on the magnetic properties of the JM-NDG and JM-DG powders was studied. Figure 3 shows the dependence of the magnetic moment per unit mass (hereafter mentioned as moment/mass) on the acid concentration. Note that the initial moment/mass of the untreated JM-NDG (153.8 emu/g) is higher than the untreated JM-DG (117.3 emu/g). This is attributed to hydrogen, which is interstitially absorbed in the Nd_2_F_14_B phase. The unit cell consequently expands, inhibiting the Fe atoms’ antiferromagnetic coupling, thus enhancing the magnetization of the hydrogenated powder (JM-NDG) [27]. 

Upon exposure to nitric acid, the measured moment/mass values of the JM-NDG powder are 152.0, 151.7, and 96.0 emu/g for 0.01, 0.1, and 1 M acid concentrations, respectively. While exposure to 0.01 and 0.1 M nitric acid inflicted only a slight decrease in the moment/mass value of the JM-NDG powder, treatment with 1 M nitric acid significantly reduced the material’s magnetic moment/mass values, indicating that the matrix Nd_2_Fe_14_B phase, which is the magnetic moment carrier in the structure of the Nd-Fe-B magnets, was severely compromised upon leaching. Results of magnetic characterization are consistent with microstructural specifics of the JM-NDG sample leached with 1 M nitric acid, i.e., damaged matrix grains, as seen in Figure 2e. Upon exposure to citric acid, the moment/mass values of the JM-NDG powder are 148.9, 158.7, and 160.1 emu/g for 0.01, 0.1, and 1 M acid concentrations, respectively. Despite the initial reduction of the moment/mass compared to the untreated powder, the values are mildly increased when acid concentration increases, contrasting the trend observed for nitric acid. Considering that citric acid selectively leaches away the Nd-rich phase, as confirmed through SEM analysis (Figure 2c), results revealed that the leaching efficiency is acid concentration-dependent. Higher citric acid concentrations leach away a higher portion of the Nd-rich phase, increasing the volume fraction of the matrix phase and raising the moment/mass value. 

Upon exposure to nitric acid, the moment/mass values of the JM-DG powder are 117.02, 118.2, and 29.4 emu/g for acid concentrations of 0.01, 0.1, and 1 M, respectively. The measured moment/mass values for citric acid are 117.0, 131.9, and 141.9 emu/g for 0.01, 0.1, and 1 M acid concentrations, respectively. The general trends in moment/mass values upon acid leaching, namely a severe decrease upon exposure to nitric and a mild increase upon exposure to citric acid, are similar for the JM-NDG and JM-DG powders, that agrees with the results of the microstructural investigation (Section 3.2).

### 3.4. ICP-MS Compositional Analysis of Non-Leached and Leached Nd-Fe-B Powders

The elemental compositions (in wt.%) of the JM-NDG and JM-DG powders before and after leaching with 1 M citric/nitric acids are presented in Table 1. For the untreated JM-NDG powder, i.e., before acid leaching, the major constituents are Fe and Nd (63.95 and 30.26 wt.%, respectively), while B is present in a smaller amount (0.88 wt.%). Other than these three primary constituents, alloying additions include Dy (0.95 wt.%), which is often added to enhance the magnets’ coercivity and high-temperature performance [27], Pr (0.56 wt.%), which is included in the Nd precursor due to the difficulty of separation and to reduce the production costs [28], Co (2.92 wt.%) to enhance the Curie temperature (T_c_) [29], and Cu (0.15 wt.%), Ga (0.18 wt.%), and Al (0.14 wt.%) to improve wetting and the grain-boundary structure and enhance coercivity [29]. The results agree with the composition of the starting alloy (Section 2.1) and neodymium magnets in the market [30,31]. Analysis revealed that the compositions of the JM-NDG and JM-DG powders are almost identical. Therefore, it is concluded that thermal degassing did not change the material’s basic chemical composition. Oxygen and hydrogen content are not reported due to the presence of those elements in the solvent and calibration solutions [32]. 

The Nd-Fe-B ternary system with a stoichiometric Nd_2_Fe_14_B composition would contain 26.70 wt.% Nd, 72.30 wt.% Fe, and 0.99 wt.% B. The total-rare earth (TRE = Nd, Pr, Dy) content in JM-NDG and JM-DG powders is 31.77 and 31.76, respectively, which is approximately 5 wt.% excess compared to the Nd_2_Fe_14_B stoichiometry, corresponding to the “extra Nd” in the Nd-rich phase. Therefore, the Nd/Fe concentration ratios before and after leaching were compared to investigate the selectivity of leaching with citric and nitric acid. Table 2 shows Nd/Fe ratio values and corresponding percent reduction relative to the Nd/Fe ratio in untreated powders, calculated for the leached samples. The Nd/Fe ratio of the JM-NDG powder decreased from 0.47 to 0.38 upon leaching with 1 M citric acid, which translates to an 18.8 % reduction, while treatment with 1 M nitric acid resulted in a negligible marginal decrease (i.e., 1.0%). A similar trend was observed for the JM-DG powder. Leaching with 1 M citric acid significantly decreased the Nd/Fe ratio from 0.47 to 0.40, i.e., by 15.9 %, while the 1 M nitric acid resulted in a negligible increase (i.e., 0.4 %). Decreased Nd/Fe values measured for both powders leached with citric acid confirm a selective behavior, i.e., preferential leaching of Nd.

Moreover, the mass fraction of Nd in the 1 M citric acid-leached JM-NDG and JM-DG samples is 26.2 and 26.8 wt.%, respectively (Table 1), which is very close to the stoichiometric Nd content of the Nd_2_Fe_14_B phase (26.7 wt.%). The results of the ICP-MS compositional analysis agree with the findings presented in Section 3.2 and Section 3.3 (microstructural and magnetic characterization, respectively), further supporting the claim that citric acid can selectively leach away the Nd-rich secondary phase.

On the other hand, the 1 M nitric acid had little effect on the Nd/Fe ratio, confirming its poor or no selectivity. Regarding the other constituent elements shown in Table 1 (Dy, Pr, B, Cu, Co, Ga, Al), no notable changes in their concentrations upon acid leaching that cannot be attributed to the fluctuations of measurement conditions or measurement errors were quantified. Considering the Nd/Fe ratio change for the JM-NDG and JM-DG powders leached with 1 M citric acid (−18.8 and −15.9, respectively), a higher proportion of Nd was leached from the former, indicating a better selectivity towards the Nd-rich phase for the non-degassed powder. However, the apparent difference in selectivity could be influenced by partial oxidation of the JM-DG powder during thermal degassing to desorb hydrogen, leading to the formation of Nd oxides which are not preferentially leached and therefore contribute to the overall Nd content in a leached sample.

### 3.5. Discussion on the Leaching Process of Nd-Fe-B Powders

Many examples in the literature target complete and selective chemical and electrochemical leaching of REEs and their extraction via various methods [20,33,34]. It was suggested by Schultz et al. [35] that there is a correlation between the corrosion rate and the electrostatic surface potential of the phases present in the structure of the Nd-Fe-B alloy. They observed that the Nd-rich phase corrodes the fastest, followed by the boron-rich phase (NdFe_4_B_4_) and then the matrix phase (Nd_2_Fe_14_B). Later, it was explained by Makarova et al. that due to the heterogeneous structure of the Nd-Fe-B alloy, selective and rapid electrochemical leaching of Nd-rich pockets surrounding the Nd_2_Fe_14_B grains happens which considering the highly reactive nature of REEs with a standard electrode potential range of −2.2 to −2.5 V, is quite reasonable [36]. The dissolution of Nd happens in two consecutive steps (Equations (1) and (2)):

Nd ⇌ Nd^2+^ + 2e^−^ E° = −2.2 V (1)

Nd ⇌ Nd^3+^ + 3e^−^ E° = −2.323 V (2)

In a more recent paper, Xu et al. suggested and proved a green method of selective electrochemical leaching to extract the Nd_2_Fe_14_B matrix phase from the EoL Nd-Fe-B magnets. However, the yield of this method is extremely low (e.g., 0.05 mg/6 h), and efforts are being made to enhance and upscale the pilot setup [18].

The present study offers an alternative chemical approach toward selective leaching of Nd-Fe-B-type materials. Diluted nitric and citric acids were considered as leaching mediums to dissolve the Nd-rich phase and obtain single-phase Nd_2_Fe_14_B powders for novel magnet making. Although a jet-milled Nd-Fe-B powder was used as a case study, the selective acid leaching process can be further optimized and adapted to recycle the Nd-Fe-B production scrap and EoL magnets. Consequently, all efforts were employed to reduce the environmental impact and costs, i.e., the leaching experiments were carried out at room temperature, without stirring or ultrasonic agitation. 

The use of nitric acid (HNO_3_) for the dissolution and extraction of REEs from secondary sources has been thoroughly investigated in the literature [37,38,39]. The leaching mechanism of Nd-Fe-B in a diluted HNO_3_ solution is explained by Equation (3) [40]:

NdFeB + 2HNO_3_ + 2H_2_O → Nd^3+^ + NO_3_^−^ + Fe^3+^ (Fe^2+^) + BO_3_ ^3−^ + NO_2_ + 6H^+^ (3)

Citric acid is an organic acid with three dissociation constant values due to having three carboxylic groups in its structure, being pKa1 = 3.13, pKa2 = 4.76, and pKa3 = 6.40 at room temperature [41]. Citric acid usually dissolves via metal chelation through the formation of a soluble metal-ligand complex or by displacement of metal ions with hydronium ions [42]. Investigation of aqueous complexation of citrate with neodymium (III) revealed the following equilibrium (Equation (4)) based on which the formation of Nd^3+^ with citrate counterion takes place:

nNd^3+^ + jH^+^ + kCit^3−^ ⇆ Nd_k_H_j_Cit_k_ ^(3n+j−3k)^ (4)

Other literature reported the formation of Nd(Cit)_2_^3−^ as the favorable product within the pH range of 5.4–6.4 [43]. 

Based on our observations, 1 M HNO_3_ attacks both the Nd-rich and the Nd_2_Fe_14_B matrix phase (depicted in Figure 2e, f), making it unsuitable for the task of selective leaching. On the other hand, citric acid selectively dissolves the Nd-rich phase. Further analysis of the kinetics of dissolution was considered out of the scope of this study. The difference in the selectivity towards the Nd-rich phase between the nitric and citric acids can be correlated with their respective pKa values. Compared to nitric acid with pKa = −1.38, citric acid with pKa1 = 3.13 is much weaker. Correspondingly, citric acid preferentially leaches away the Nd-rich phase, which is more prone to oxidation than the matrix phase [44,45,46]. Importantly, utilizing organic acids requires much fewer purification and detoxification steps and has a much smaller toll on the environment than strong mineral acids [21,39]. Finally, due to the simplicity of the acid leaching approach, the process can be upgraded to industrial scales. 

### 3.6. Discussion on the Environmental and Economic Impact

The supply risk and environmental and economic aspects are key factors that make an adaptation of recycling Nd-Fe-B magnets prudent [47]. According to the literature, the neodymium and dysprosium in use globally are five times their annual extraction rate [48], which further emphasizes this potential. The most investigated routes for recycling Nd-Fe-B magnets are the direct reuse, waste-to-REE, waste-to-alloy, and magnet-to-magnet approaches. Except for direct reuse, hydrometallurgy, pyrometallurgy, hydrogen decrepitation, gas-phase extraction, etc., are utilized for recycling [30]. Research shows that hydrometallurgy is less harmful to the environment than pyrometallurgy due to lesser energy consumption and reduced material loss.

Nonetheless, compared to hydrogen decrepitation, they produce more chemical waste and use more chemicals (pyrometallurgy) or more energy (Hydrometallurgy) [30]. Producing 1000 tons of Nd-Fe-B magnets from scratch requires 32,519,287 kWh/year of energy, while only 2,940,260 kWh/year is needed for conventional recycling, i.e., a 91% saving in energy [49]. From the environmental perspective, preliminary calculations show a reduction of 11 tons of CO_2_ emissions and ~4 tons of chemicals per ton of magnets produced via recycling while reducing contaminants, radioactive waste, etc. [30,49]. This study takes the product of the hydrogen decrepitation process, a coarse Nd-Fe-B powder with disfigured and inhomogeneous Nd-rich phase, which can easily oxidize or pick up contaminants, and effectively removes them, leaving behind intact Nd-Fe-B grains. In this process, we eliminate the steps requiring a significant amount of chemicals and/or energy via current conventional recycling methods, replacing it with a much more environmentally friendly and economical process. Moreover, this process improves the efficiency of the state-of-the-art techniques by conserving the Nd-Fe-B grains (the 2:14:1 phase) and their magnetic properties. It also enables the investigation and incorporation of newly developed grain-boundary phases for optimization and adjustment of magnetic properties to the need of the consumer party or application.

## 4. Conclusions

This study explores the concept of acid leaching using citric and nitric acid on Nd-Fe-B-type multiphase powders, intending to selectively dissolve the Nd-rich phase and extract the Nd_2_Fe_14_B grains. A microcrystalline jet-milled powder, the precursor to sintered Nd-Fe-B magnets, represents an appropriate case study. The same approach can be used for recycling Nd-Fe-B production scrap and end-of-life magnets. The effect of hydrogen present in the material was investigated based on the trend of the magnetic moment/mass of the recovered powder after leaching with citric and nitric acids and was found not to influence the leaching efficiency that validates the compatibility of the selective acid leaching with citric acid approach with the existing technologies, namely hydrogen processing of magnetic scrap. ICP-MS analysis showed that the concentration of the Nd in the recovered powder after leaching with 1M citric acid was reduced to 26.22 and 26.76 for JM-NDG and JM-DG samples, respectively. These values completely agree with the theoretical values of the Nd in the structure of the Nd_2_Fe_14_B phase. The magnetic measurements also illustrated that the magnetic moment/mass of the JM-NDG and JM-DG samples were increased after leaching with 1 M citric acid to 141.9 and 160.1 emu/g, respectively. Results of the SEM microstructural investigation and ICP-MS compositional analysis and magnetic measurements of the leached Nd-Fe-B powders confirm that citric acid can indeed selectively leach away the Nd-rich secondary phase while preserving the Nd_2_Fe_14_B matrix phase with respect to its magnetic moment/mass values. Exposure to nitric acid results in a structurally damaged Nd_2_Fe_14_B matrix phase, as evidenced by the SEM investigations and severely reduced magnetic moment/mass values of the powder. The moment/mass values of 96.0 and 29.4 emu/g for JM-NDG and JM-DG samples were recorded after leaching with nitric acid, respectively. Using mild organic acids such as citric acid, in our case, makes the proposed selective acid leaching efficient for removing the Nd-rich phase selectively and environmentally friendly, as the leaching medium can be easily neutralized after the reaction is completed. Moreover, the leaching process can be upscaled and is therefore suitable for industrial-scale magnet recycling.

## Figures and Tables

**Table 1:** Elemental composition of the untreated JM-NDG and JM-DG powders and samples leached with 1 M citric and nitric acid.

Sample	JM-NDG		JM-NDG-1M CIT		JM-NDG-1M-NIT		JM-DG		JM-DG-1M-CIT		JM-DG-1M-NIT	
Element	Average	Stdev	Average	Stdev	Average	Stdev	Average	Stdev	Average	Stdev	Average	Stdev
Nd	30.26	0.49	26.22	1.05	30.12	0.75	30.28	0.53	26.76	1.90	30.35	0.61
Dy	0.95	0.01	0.98	0.01	0.93	0.03	0.93	0.01	1.00	0.04	0.95	0.02
Fe	63.95	0.86	68.21	1.89	64.33	0.98	64.08	1.00	67.34	5.60	63.94	1.41
Pr	0.56	0.01	0.45	0.01	0.54	0.02	0.55	0.01	0.49	0.02	0.56	0.01
B	0.88	0.02	0.89	0.01	0.85	0.02	0.86	0.01	0.90	0.02	0.88	0.02
Cu	0.15	0.00	0.17	0.00	0.14	0.00	0.14	0.00	0.15	0.01	0.15	0.00
Co	2.92	0.04	2.84	0.02	2.78	0.09	2.84	0.03	3.04	0.15	2.87	0.03
Ga	0.18	0.00	0.09	0.00	0.17	0.01	0.17	0.00	0.15	0.01	0.17	0.00
Al	0.14	0.00	0.15	0.01	0.14	0.00	0.14	0.00	0.16	0.02	0.14	0.00
Total	100	1.4	100	3	100	2	100	2	100	8	100	2

**Table 2:** The Nd/Fe ratios and their corresponding percent reductions in wt.%.

Powder Type	Nd/Fe	Percent Reduction
JM-NDG	0.47	N/A
JM-NDG—1M citric acid leached	0.38	−18.8
JM-NDG—1M nitric acid leached	0.47	−1.0
JM-DG	0.47	N/A
JM-DG—1M citric acid leached	0.40	−15.9
JM-DG—1M nitric acid leached	0.47	+0.4

## Data Availability

The data presented in this study are available on request from the corresponding author. The data are not publicly available due to privacy reasons.

## Supplementary Materials

The following supporting information can be downloaded at: https://www.mdpi.com/article/10.3390/ma16145181/s1, Table S1. ICP-MS instrumental parameters used to determine element concentrations in magnet samples. Figure S1. Evolution of pressure with temperature up to 1000 °C for JM-NDG powder.
