# Supplementary material for: Environmentally Friendly Approach for Nd2Fe14B Magnetic Phase Extraction by Selective Chemical Leaching: A Proof-of-Concept Study
Source: Materials (Basel). 2023 Jul 23;16(14):5181. doi: 10.3390/ma16145181 (PMC10384185; doi:10.3390/ma16145181)
Supplement: Supplementary file 1 [file materials-16-05181-s001.zip › materials-2459048-supplementary.pdf]

**Table S1.** ICP-MS instrumental parameters used to determine element concentrations in magnet samples.

| ***Parameter*** | ***Type/Value*** |
|---|---|
| *Plasma condition* | |
| Forward power | 1550 W |
| Plasma gas flow | 15.0 L $min^{-1}$ |
| Carrier gas flow | 1.05 L $min^{-1}$ |
| Sample depth | 8 mm |
| *Cell parameters* | |
| He gas flow | 4.3 mL $min^{-1}$ |
| Octopole bias | -18.0 V |
| Octopole RF | 200 V |
| Energy discrimination | 5.0 V |
| *Data acquisition parameters* | |
| Isotopes monitored | $^{11}B$, $^{27}Al$, $^{56}Fe$, $^{59}Co$, $^{63}Cu$, $^{69}Ga$, $^{141}Pr$, $^{146}Nd$,$^{163}Dy$ |
| Isotopes of internal standards | $^{103}Rh$, $^{193}Ir$ |
| Integration time per isotope | 0.1 s |

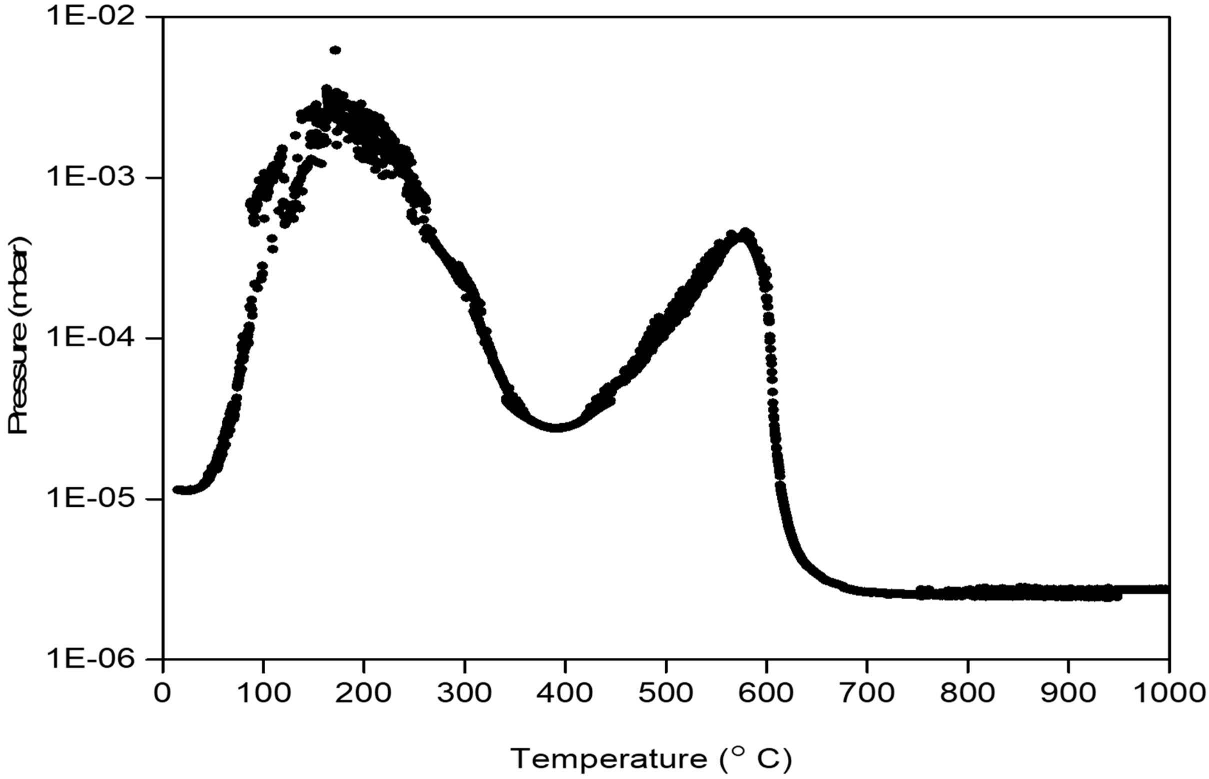


**Figure S1.** Evolution of pressure with temperature up to 1000 °C for JM-NDG powder.
